# Investigating potential targets of Wulingsan in diabetic nephropathy through network pharmacology and experimental validation

**DOI:** 10.3389/fmolb.2025.1647796

**Published:** 2025-07-29

**Authors:** Xicheng Hu, Zhen Wang, Liyan Zhang

**Affiliations:** ^1^ Department of Traditional Chinese Medicine, General Hospital of Ningxia Medical University, Yinchuan, Ningxia, China; ^2^ Department of Integrated Traditional Chinese and Western Medicine, Affiliated Hospital of Hebei University, Baoding, Hebei, China

**Keywords:** Wulingsan, diabetic nephropathy, network pharmacology, molecular docking, experimental validation

## Abstract

**Background:**

Diabetic Nephropathy (DN), a major microvascular complication of diabetes, poses challenges for current treatments to effectively delay its progression. Wulingsan (WLS), a traditional Chinese medicine formula, possesses potential for regulating water-fluid metabolism, and exhibits anti-inflammatory and antioxidant properties, yet its multi-target mechanism in treating DN remains unclear. This study aims to systematically elucidate the molecular mechanisms of WLS in the treatment of DN through network pharmacology, molecular docking, and *in vitro* experiments.

**Methods:**

Active ingredients of WLS and their targets were screened using the TCMSP database, while DN-related targets were obtained from the GeneCards and OMIM databases to construct an “ingredient-target-disease” network. GO and KEGG pathway enrichment analyses were performed using DAVID to identify key biological processes and signaling pathways. A protein-protein interaction (PPI) network was constructed via the STRING database, and key targets were screened using the CytoHubba plugin. Subsequently, molecular docking and molecular dynamics simulations were conducted to validate the binding affinity and stability of active ingredients with key targets. *In vitro*, a high glucose-induced HK-2 cell model was employed, and the effects of WLS on cell viability and cell cycle were assessed using CCK-8 assays and flow cytometry, respectively.

**Results:**

The study screened and identified SRC, AKT1, TNF, ESR1, and HSP90AA1 as key targets for the treatment of DN. KEGG enrichment analysis revealed that WLS primarily regulates signaling pathways such as PI3K-Akt and MAPK, which are closely associated with inflammation, oxidative stress, and fibrosis. Molecular docking indicated that active ingredients (β-caryophyllene, alisol C) exhibited binding energies below −5.0 kcal/mol with key targets (TNF, HSP90AA1), and molecular dynamics simulations further validated their binding stability. *In vitro* experiments demonstrated that WLS significantly inhibited the proliferation of high glucose-induced HK-2 cells (P < 0.01) and induced G2/M phase cell cycle arrest (P < 0.01).

**Conclusion:**

Wulingsan alleviates the progression of Diabetic Nephropathy by its multiple active ingredients acting synergistically on key targets such as SRC, AKT1, and TNF, thereby regulating PI3K-Akt and MAPK signaling pathways to inhibit inflammation, oxidative stress, and fibrosis. This research provides a theoretical basis for the clinical application of WLS, and its therapeutic efficacy warrants further verification through future *in vivo* experiments.

## Graphical Abstract

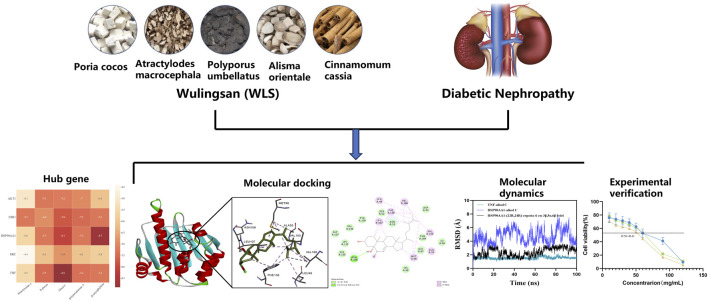

## Introduction

Diabetic nephropathy (DN) is a severe microvascular complication of diabetes mellitus and a leading cause of end-stage renal disease (ESRD) worldwide (Liu et al., 2010). It is characterized by progressive kidney damage, including albuminuria, glomerulosclerosis, and interstitial fibrosis, which ultimately result in renal failure (Kong et al., 2021). The pathogenesis of DN involves a complex interplay of metabolic disturbances, oxidative stress, chronic inflammation, and fibrosis, leading to impaired renal function and irreversible kidney injury (Guo et al., 2017). Current therapeutic strategies, including angiotensin-converting enzyme (ACE) inhibitors and sodium-glucose cotransporter-2 (SGLT2) inhibitors, primarily focus on managing symptoms by controlling blood glucose levels and reducing renal hemodynamic stress (Huang et al., 2018). However, these interventions fail to effectively halt or reverse the progression of DN, and their long-term use is associated with adverse effects such as hypotension, electrolyte imbalances, and an increased risk of infections (Yang et al., 2023). Consequently, there is a pressing need for safer and more efficacious treatment approaches that target the underlying mechanisms of DN and provide long-term renal protection.

Traditional Chinese medicine (TCM), with its multi-component, multi-target approach, offers a promising alternative for managing complex diseases like DN. Wulingsan (WLS), a classic TCM formula composed of Polyporus umbellatus, Alisma orientale, Poria cocos, Atractylodes macrocephala, and Cinnamomum cassia, has been historically used to regulate fluid metabolism and alleviate edema—a hallmark of DN. In TCM theory, WLS is primarily used to promote diuresis, drain dampness, strengthen the spleen, and warm the yang, which align with the pathological features of DN, including fluid retention and metabolic dysfunction (Chen et al., 2021).

Modern pharmacological studies have revealed its diuretic, anti-inflammatory, antifibrotic, and antioxidant properties, supporting its potential role in DN management (Paquissi and Abensur, 2021). For instance, Alisma orientale extract mitigates renal fibrosis by suppressing the TGF-β/Smad signaling pathway, while Poria cocos polysaccharides exhibit renoprotective effects through modulation of oxidative stress and inflammatory cytokines (Chen et al., 2020). Moreover, Polyporus umbellatus has been reported to enhance autophagy and reduce renal tubular injury, while Atractylodes macrocephala contributes to immune regulation and gut microbiota modulation, which are increasingly recognized as key factors in DN pathogenesis (Zhu et al., 2022). Cinnamomum cassia, rich in cinnamaldehyde, has been found to exert hypoglycemic and anti-inflammatory effects by modulating the NF-κB and Nrf2 pathways, which are crucial in DN progression (Guo et al., 2022). Recent studies have also demonstrated that WLS may protect against renal damage in DN by regulating apoptosis-related proteins, including the Caspases family and BCL2 protein family, thereby reducing kidney cell injury and delaying the progression of DN (Feng et al., 2021). Despite these findings, the systemic mechanisms underlying WLS’s therapeutic efficacy in DN remain poorly elucidated, limiting its clinical application.

Network pharmacology represents a transformative approach in drug discovery, offering profound insights into the complex mechanisms of TCM. By mapping the interactions between bioactive compounds, target proteins, and disease-related pathways, network pharmacology allows for a deeper understanding of the holistic therapeutic effects of TCM formulas, such as Wulingsan (Zhao et al., 2023). This approach goes beyond individual compounds, integrating the multifaceted interactions within herbal ingredients, molecular targets, and the biological systems they influence. By constructing comprehensive interaction networks, network pharmacology sheds light on the diverse molecular mechanisms through which TCM formulas like Wulingsan exert their therapeutic benefits (Ren et al., 2023).

Molecular docking, a crucial technique in drug discovery, further enhances this understanding by simulating the binding interactions between bioactive compounds and their target proteins. This enables a more precise identification of the key molecular targets involved in the therapeutic actions of Wulingsan, providing valuable insights into its potential efficacy (Gao et al., 2025; Zhao et al., 2025). Together, these tools offer a comprehensive and integrated framework for elucidating the complex, multi-target nature of TCM treatments.

In this study, we utilized a network pharmacology approach to map the targets of WLS with those associated with DN. The resulting interactions were imported into the STRING database, where a protein-protein interaction (PPI) network was constructed under the settings of Multiple proteins and *Homo sapiens*. This network, consisting of 269 nodes and 4,201 edges, reflects the *in vivo* mechanisms through which WLS exerts its therapeutic effects on DN. To further identify the key therapeutic targets, we applied eight algorithms (Betweenness, Closeness, Degree, EPC, MCC, MNC, Radiality, and Stress) from the CytoHubba plugin in Cytoscape. The intersection of these algorithms led to the identification of five core targets: SRC, AKT1, TNF, ESR1, and HSP90AA1. These targets represent central nodes in the therapeutic network of WLS for DN. Additionally, to validate the interaction between these core targets and bioactive compounds within WLS, molecular docking and molecular dynamics simulations were performed. The results from these computational methods provided further insight into the binding affinities and stability of key compound-target interactions, confirming the molecular mechanisms underlying the therapeutic potential of WLS in DN.

## Materials and methods

### Screening of active ingredients of WLS and related targets

The active compounds of WLS were identified using the Traditional Chinese Medicine Systems Pharmacology Database and Analysis Platform (TCMSP, http://tcmspw.com/tcmsp.php), which provides detailed information on the absorption, distribution, metabolism, and excretion (ADME) characteristics, as well as potential molecular targets of traditional Chinese medicine constituents. Compounds were screened based on established pharmacokinetic parameters, with oral bioavailability (OB) ≥ 30% and drug-likeness (DL) ≥ 0.18 as the primary criteria (Ru et al., 2014). For components derived from Cinnamomum cassia, a lower DL threshold of ≥0.12 was applied in accordance with prior studies (Liu et al., 2020).

**FIGURE 1:**
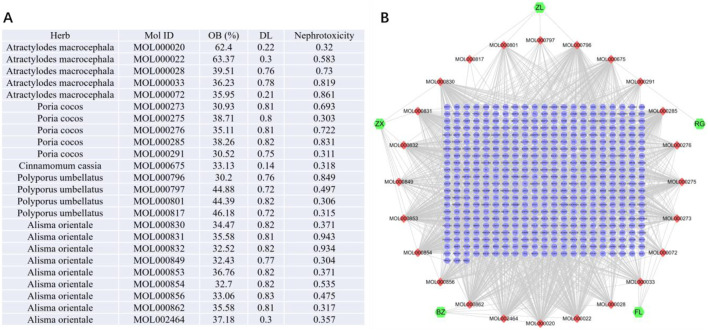
Component-target network of WLS targets. **(A,B)** The nodes represent various compounds, with red nodes indicating active compounds and green nodes representing key targets. The edges between the nodes depict the interactions between these compounds and targets.

A total of 24 active ingredients from the five herbs of Wulingsan (WLS) were identified. These included five compounds from Atractylodes macrocephala, five from Poria cocos, one from Cinnamomum cassia, four from Polyporus umbellatus, and nine from Alisma orientale. The component-target network analysis revealed that several compounds, including MOL000022, MOL000796, MOL000853, MOL000854, and MOL000862, had high degree values, indicating their potential importance in the therapeutic effects of WLS (Figure 1).

The SMILES (Simplified Molecular Input Line Entry System) notations for each compound were retrieved from the PubChem database (https://pubchem.ncbi.nlm.nih.gov/) to facilitate computational analysis. Nephrotoxicity predictions were performed using the ADMETlab 3.0 platform (https://admetlab3.scbdd.com/server/evaluation), which integrates multiple models to assess absorption, distribution, metabolism, excretion, and toxicity (ADMET) profiles. The potential targets of these components were predicted using the Swiss Target Prediction platform (http://www.swisstargetprediction.ch/) by inputting the SMILES codes. Targets with a probability score >0 were selected for subsequent analysis.

### Functional enrichment analysis of component targets

The identified targets of WLS were imported into the Database for Annotation, Visualization and Integrated Discovery (DAVID, https://david.ncifcrf.gov/) for functional enrichment analysis. The analysis was performed with *Homo sapiens* as the species background and a significance threshold of P < 0.05. Gene Ontology (GO) analysis, including Biological Processes (BP), Cellular Components (CC), and Molecular Functions (MF), as well as Kyoto Encyclopedia of Genes and Genomes (KEGG) pathway analysis, was conducted to explore the potential biological functions and pathways associated with the WLS targets. The results were visualized as bubble charts based on P-values.

### Protein-protein interaction (PPI) network construction and core target identification

The common targets between WLS and DN were imported into the STRING database to construct a protein-protein interaction (PPI) network. The network was built using the “Multiple proteins” and “*Homo sapiens*” settings to reflect the *in vivo* interaction network of WLS in treating DN. To identify the key therapeutic targets, eight algorithms (Betweenness, Closeness, Degree, EPC, MCC, MNC, Radiality, and Stress) from the CytoHubba plugin in Cytoscape were applied to calculate the importance of each node in the network. The intersection of the top-ranked targets from all eight algorithms was considered as the core targets of WLS for DN treatment.

### Molecular docking

Molecular docking was conducted to explore the interactions between hub target proteins and key active compounds. The protein structures were obtained from the RCSB Protein Data Bank (PDB). Preparatory steps, including removing water molecules, separating the native ligand, adding hydrogen atoms, repairing broken chains, and calculating protein charges, were performed using PyMOL and AutoDock (version 1.5.6). The active compound structures were retrieved from ZINC and saved in MOL2 format, then converted to PDBQT using Open Babel. Docking was performed with AutoDock 1.5.6, and results were analyzed using PyMOL. Docking poses with binding energies below −5.0 kcal/mol were considered favorable, and those below −7.0 kcal/mol indicated strong affinity. Poses that met both the binding energy and hydrogen bond formation criteria were selected for further analysis.

### Molecular dynamics

A 100 ns molecular dynamics (MD) simulation of the protein–ligand complex was conducted using Gromacs 2023. The protein was modeled with the CHARMM 36 force field parameters (Li and Wang, 2019), while the ligand topology was constructed using the GAFF2 force field parameters. The complex was placed in a cubic box under periodic boundary conditions, and the system was solvated with water molecules using the TIP3P water model (Wang et al., 2019).

Electrostatic interactions were computed using the Particle Mesh Ewald (PME) method, while neighbor searching was handled via the Verlet algorithm. After system preparation, 100,000 equilibration steps were performed for both constant-volume (NVT) and constant-pressure (NPT) ensembles, with a coupling constant of 0.1 ps over a 100-ps period. Both van der Waals and Coulomb interactions were calculated using a 1.0 nm cutoff. Finally, the production MD simulation was run for 100 ns at 300 K and 1 bar using Gromacs 2023 (Islam et al., 2018).

### Cell culture

Human renal proximal tubular epithelial cells (HK-2, Cat. No. GNHu47) were procured from the China Center for Type Culture Collection (CCTCC). HK-2 cells were maintained in Keratinocyte Serum-Free Medium (K-SFM; Invitrogen, Cat. No. 17005042) supplemented with 10% fetal bovine serum (FBS) and Gentamicin/Amphotericin Solution (Gibco, Cat. No. R01510). Cultures were maintained in a humidified incubator at 37°C with an atmosphere of 5% CO_2_. Cell confluence, typically reaching 70%–80%, was monitored microscopically prior to experimentation or passaging.

### CCK-8 assay

The formula granules of WLS, consisting of Polyporus (9 g), Poria cocos (9 g), Alisma orientale (12 g), Atractylodes macrocephala (6 g), and Cinnamomum cassia (9 g), were obtained from Beijing Sanofi Pharmaceutical Co., Ltd. (Beijing, China). The granules were dissolved in distilled water at 100°C for preparation.

The half-maximal inhibitory concentration (IC_50_) of WLS was determined using a Cell Counting Kit-8 (CCK-8) assay. HK-2 cells were seeded into 96-well plates at a density of 1 × 105 cells/well, with five replicate wells per group. Experimental groups comprised: a control group (CON) cultured in complete cell culture medium; a model group (MOD) cultured in medium supplemented with 70 mmol/L high glucose; and WLS treatment groups exposed to serially diluted concentrations of WLS in high-glucose medium. Cells were incubated with these treatments for 24, 48, and 72 h. Following the respective incubation periods, 10 µL of CCK-8 solution was added to each well (containing 100 µL of culture medium), and the plates were incubated for an additional 1 h at 37°C. The absorbance, indicative of cell viability, was measured at an optical density (OD) of 450 nm using a microplate reader for subsequent statistical analysis and IC_50_ calculation. All *in vitro* experiments were performed in triplicate (*n* = 3) independent biological replicates.

### Flow cytometry analysis of cell cycle progression

The impact of WLS on cell cycle progression in HK-2 cells under high-glucose conditions was evaluated by flow cytometry. Logarithmically growing HK-2 cells were allocated to five experimental groups: (1) CON: cultured in complete cell culture medium; (2) MOD: cultured in 70 mmol/L high-glucose medium; (3) Low-dose WLS: high-glucose medium supplemented with 40 mg/mL WLS; (4) Medium-dose WLS: high-glucose medium supplemented with 50 mg/mL WLS; and (5) High-dose WLS: high-glucose medium supplemented with 60 mg/mL WLS. Cells were processed for cell cycle analysis according to the manufacturer’s instructions of the relevant kit. The distribution of cells in different phases of the cell cycle (G0/G1, S, G2/M) was quantified using a flow cytometer, followed by statistical analysis. All *in vitro* experiments were performed in triplicate (*n* = 3) independent biological replicates.

### Statistical analysis

All statistical analyses were conducted using GraphPad Prism 8.0 software (GraphPad Software, San Diego, CA, United States). Intergroup comparisons were performed using one-way analysis of variance (ANOVA). P-value <0.05 was considered indicative of statistical significance.

## Results

### Active ingredients of WLS and their targets

Based on the screening criteria (OB ≥ 30%, DL ≥ 0.18, nephrotoxicity ≥0.3), a total of 24 active ingredients from the five herbs of Wulingsan (WLS) were identified. These included five compounds from Atractylodes macrocephala, five from Poria cocos, one from Cinnamomum cassia, four from Polyporus umbellatus, and nine from Alisma orientale. The component-target network analysis revealed that several compounds, including MOL000022, MOL000796, MOL000853, MOL000854, and MOL000862, had high degree values, indicating their potential importance in the therapeutic effects of WLS (Figure 1).

### Functional enrichment analysis of WLS targets

**FIGURE 2:**
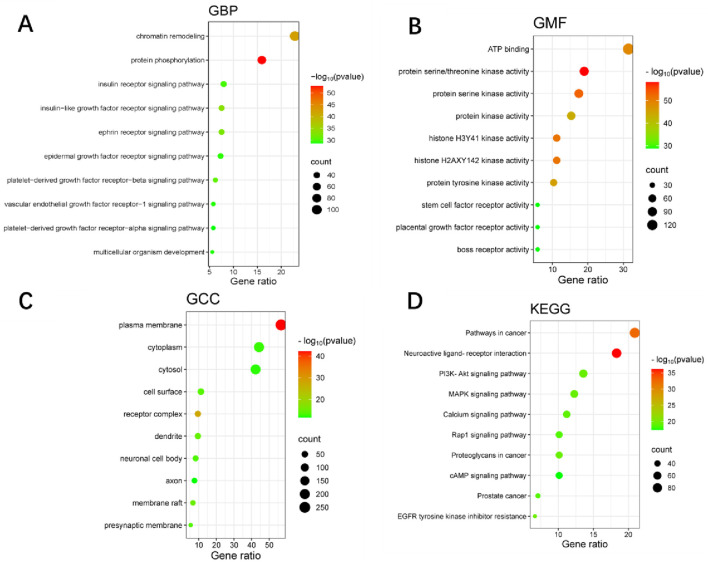
GO and KEGG pathway enrichment analyses of WLS targets. **(A)** Shows enriched biological processes. **(B)** Illustrates molecular functions. **(C)** Depicts cellular components. **(D)** Presents KEGG pathways. In each panel, the color intensity represents–log_10_(p-value), while the dot size reflects the Gene Count.

The GO and KEGG pathway enrichment analysis of WLS targets identified 804 biological processes, 100 cellular components, and 241 molecular functions. Additionally, 161 KEGG signaling pathways were identified. The most significantly enriched biological processes included chromatin remodeling, protein phosphorylation, and various receptor signaling pathways, such as insulin receptor and epidermal growth factor receptor signaling. Among the molecular functions, ATP binding and protein kinase activities were the most prominent. Cellular component enrichment highlighted the plasma membrane, cytoplasm, and receptor complexes. Notably, the most significantly enriched KEGG pathways included neuroactive ligand-receptor interaction, pathways in cancer, EGFR tyrosine kinase inhibitor resistance, PI3K-Akt signaling pathway, MAPK signaling pathway, and calcium signaling pathway (Figure 2).

### Disease target prediction

**FIGURE 3:**
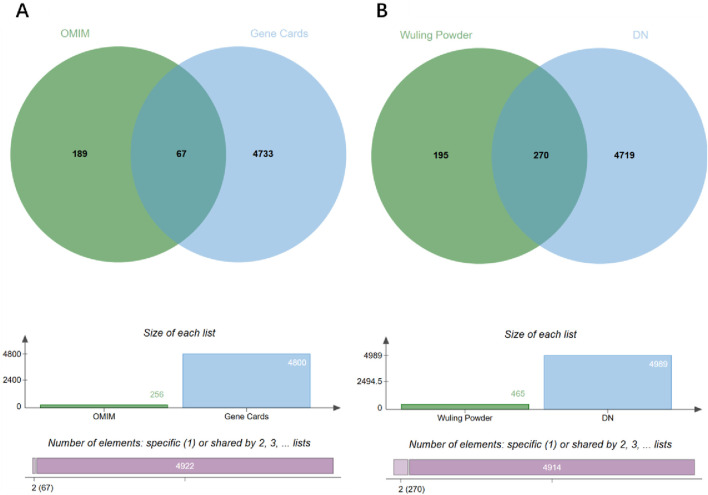
Venn diagram analysis of disease targets and WLS targets. **(A)** Venn diagram of disease targets. **(B)** Venn diagram of intersection targets.

Using the keyword “diabetic nephropathy,” DN-related targets were retrieved from the GeneCards database (https://www.genecards.org/) with a relevance score threshold of ≥10, and from the OMIM database (https://omim.org/), where only genes explicitly annotated as associated with DN were selected, resulting in 4,800 and 256 targets respectively. After removing duplicates, a total of 4,989 unique disease-related targets were obtained. The gene datasets for diabetic nephropathy-related targets and Wulingsan-related targets were then imported into the online bioinformatics visualization platform (http://www.bioinformatics.com.cn) to determine the intersecting genes between the two datasets. This analysis yielded 270 overlapping targets, as illustrated in Figure 3.

### Enrichment analysis of common targets in WLS and DN

**FIGURE 4:**
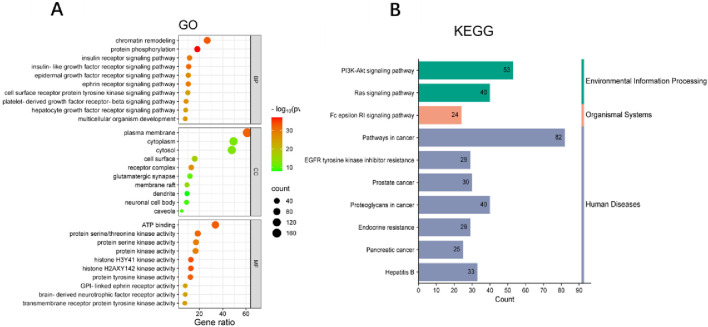
Enrichment analysis of common Targets in WLS and DN. **(A)** GO enrichment analysis of the intersection targets between Wulingsan and diabetic nephropathy. **(B)** KEGG pathway enrichment analysis of the same targets. In each panel, the color intensity represents–log_10_(p-value), while the dot size reflects the Gene Count.

The common targets between Wulingsan (WLS) and diabetic nephropathy (DN) were mapped and the resulting dataset was imported into the online DAVID tool, with the species set as *Homo sapiens* and a significance threshold of P < 0.05. GO enrichment analysis was performed by selecting the GO categories of Biological Processes, Molecular Functions, and Cellular Components, as well as KEGG pathway analysis. The enriched GO and KEGG results were visualized by plotting their p-values. In total, the analysis identified 710 GO Biological Process terms, 94 GO Cellular Component terms, 193 GO Molecular Function terms, and 156 KEGG signaling pathways. Notably, the significantly enriched KEGG pathways included Pathways in cancer, EGFR tyrosine kinase inhibitor resistance, PI3K-Akt signaling pathway, Prostate cancer, Proteoglycans in cancer, Endocrine resistance, Ras signaling pathway, Pancreatic cancer, Fc epsilon RI signaling pathway, and Hepatitis B (Figure 4).

### Protein interaction network construction of core targets and identification of hub genes

**FIGURE 5:**
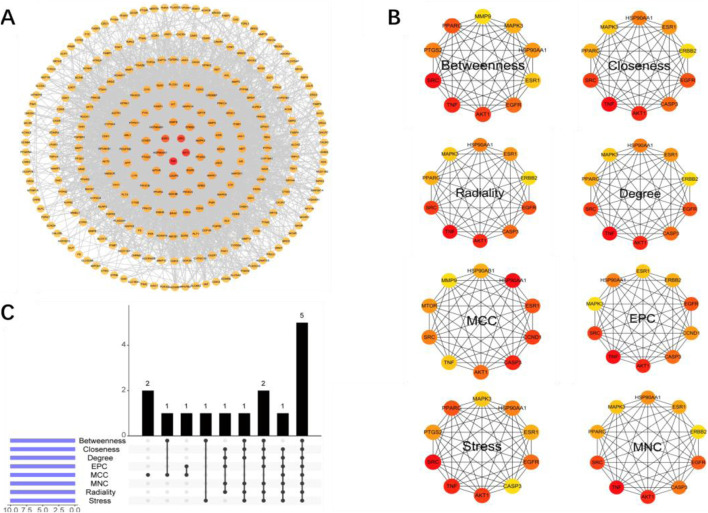
Construction of the Core Target PPI Network and Identification of Key Genes. **(A)** PPI network constructed based on core targets, illustrating the core genes involved in the intervention of diabetic nephropathy by WLS, as well as their interactions. **(B)** Top 10 key genes identified by eight different algorithms in Cytoscape (MCC, MNC, EPC, Degree, Closeness, Betweenness, Radiality, Stress). **(C)** Intersection analysis of the key genes identified by these eight algorithms.

**FIGURE 6:**
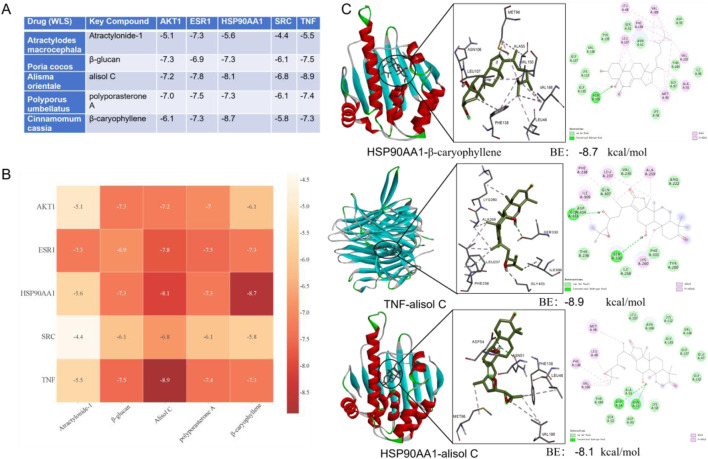
Molecular docking analysis of key natural active components of WLS with potential targets. **(A)** Presentation of docking results and binding energies between WLS active components and core target molecules; **(B)** Heatmap display of molecular docking binding energy results; **(C)** Demonstration of docking outcomes between WLS active components and core target molecules. WLS, Wulingsan.

To further identify the hub genes responsible for the therapeutic effects of Wulingsan in diabetic nephropathy, we constructed a protein-protein interaction (PPI) network for the core targets (Figure 6A) and identified key genes using eight different algorithms in Cytoscape software (MCC, MNC, EPC, Degree, Closeness, Betweenness, Radiality, and Stress). The top 10 genes from each algorithm were ranked as the key genes for that specific algorithm (Figure 6B). Among these, the MCC-key genes were SRC, CASP3, AKT1, MAPK3, TNF, ESR1, ERBB2, HSP90AA1,EGFR, PPARG; EPC-key genes were SRC, CASP3, AKT1, MAPK3, TNF, ESR1, ERBB2, HSP90AA1, EGFR, CCND1; Degree-key genes were SRC, CASP3, AKT1, MAPK3, TNF, ESR1, ERBB2, HSP90AA1, EGFR, PPARG; Closeness-key genes were SRC, CASP3, AKT1, MAPK3, TNF, ESR1, ERBB2, HSP90AA1, EGFR, PPARG; Betweenness-key genes were SRC, AKT1, PTGS2, MAPK3, TNF, ESR1, HSP90AA1, EGFR, PPARG, MMP9; Radiality-key genes were SRC, AKT1, TNF, ESR1, HSP90AA1; Stress-key genes were SRC, CASP3, AKT1, MAPK3, TNF, ESR1, ERBB2, HSP90AA1, EGFR, PPARG. Finally, the key genes identified by these eight algorithms were intersected, resulting in the identification of five core genes: SRC, AKT1, TNF, ESR1, and HSP90AA1 (Figures 5A,C). These core genes were defined as the hub genes responsible for the effects of WLS in diabetic nephropathy.

### Molecular docking

Molecular docking was performed between key compounds (Atractylonide-I, β-glucan, alisol C, polyporasterone A, and β-caryophyllene) from medicinal herbs (*Atractylodes macrocephala*, *Poria cocos*, *Alisma orientale*, *Polyporus umbellatus*, and *Cinnamomum cassia*) and target proteins AKT1, ESR1, HSP90AA1, SRC, and TNF. Docking results, including scores (Figure 6A), a binding affinity heatmap (Figure 6B), and 2D/3D interaction diagrams (Figure 6C), were assessed using docking scores. Scores below −4.25 kcal/mol indicated binding, and below −5.0 kcal/mol indicated strong binding. Many compounds showed strong binding energies (below −5.0 kcal/mol). TNF-alisol C exhibited the most favorable binding at −8.9 kcal/mol, with HSP90AA1-β-caryophyllene (−8.7 kcal/mol) and HSP90AA1-alisol C (−8.1 kcal/mol) also being notable (Figure 6C). All interactions presented in Figure A had binding energies below −4.4 kcal/mol.

### Molecular dynamics simulation analysis

**FIGURE 7:**
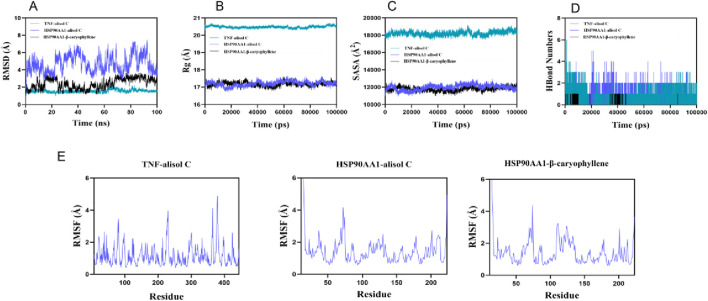
Molecular dynamics simulation diagram. **(A)** the RMSD curves; **(B)** the Rg curves; **(C)** the SASA curves; **(D)** the fluctuation curves of the number of hydrogen bonds; **(E)** the RMSF curves.

Root means square deviation (RMSD) was used to assess system equilibration. The three complex systems (TNF-alisol C, HSP90AA1-alisol C, and HSP90AA1-β-caryophyllene) reached equilibrium after 10 ns, 95 ns, and 70 ns, respectively, with their RMSD values ultimately fluctuating around approximately 6.2 Å (Figure 7A). Among these, the TNF-alisol C system exhibited a lower overall RMSD value, indicating higher stability when bound to the TNF target protein. The radius of gyration (Rg) and solvent accessible surface area (SASA) of these three systems showed slight fluctuations throughout the simulation, suggesting that conformational changes occurred (Figures 7B,C).

Hydrogen bond analysis (Figure 7D) revealed that the TNF-alisol C system predominantly formed approximately three hydrogen bonds (range 0–6), and the HSP90AA1-alisol C system also mainly formed about three hydrogen bonds (range 0–5), while the HSP90AA1-β-caryophyllene system formed fewer hydrogen bonds (mainly ∼1, range 0–1). This indicates that hydrogen bond interactions were present in all systems. Furthermore, the root mean square fluctuation (RMSF) values of amino acid residues in all systems were relatively low (mostly below 3 Å), indicating low flexibility and high overall stability (Figure 7E). All three complexes demonstrated stable binding. Particularly, the TNF-alisol C complex, owing to its lower RMSD value and favorable hydrogen bonding interactions, exhibited excellent binding characteristics with the TNF target protein.

### Experimental verification of cell viability and cell cycle

**FIGURE 8:**
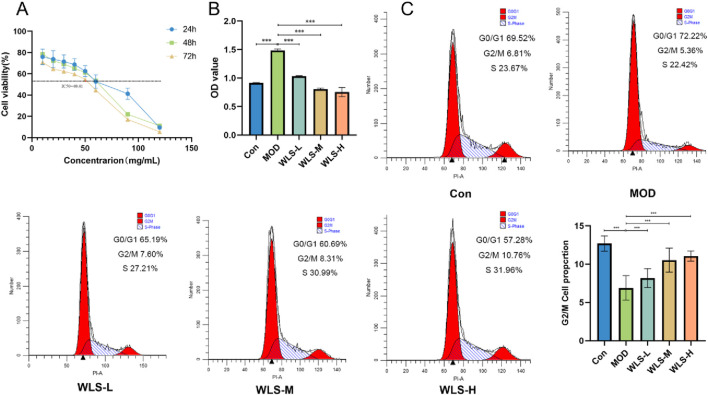
Verification of cell viability and cell cycle. **(A)** Impact of WLS on the IC_50_ of HK-2 cells; **(B)** Influence of WLS on the activity of HK-2 cells; **(C)** Effect of WLS on the cell cycle of HK-2 cells (***P < 0.001).

We measured HK-2 cell viability at 24, 48, and 72 h to determine the half-maximal inhibitory concentration (IC50) of WLS (Figure 8A). The 72-h time point was selected for further experiments as cell growth was optimal then. Under high glucose conditions, compared to the control group (CON), high glucose treatment (MOD group) significantly increased HK-2 cell viability (P < 0.05, and this increase was robust, P < 0.01; Figure 8B). In contrast, WLS treatment significantly reduced HK-2 cell viability compared to the MOD group, and this effect was dose-dependent, meaning higher WLS doses caused greater reductions in viability (P < 0.01; Figure 8B). To investigate the mechanism of WLS action, flow cytometry was used to examine changes in the HK-2 cell cycle after 24 h of WLS treatment under high-glucose conditions. The results showed that, compared to control cells (CON), high-glucose treatment (MOD group) decreased the proportion of cells in the G0/G1 phase and increased the proportion of cells in the S and G2/M phases (P < 0.01 for all changes; Figure 8C). WLS treatment, however, significantly altered this cell cycle distribution pattern: compared to the MOD group, WLS led to an increased percentage of cells in both the G0/G1 and G2/M phases (P < 0.01). These results indicate that WLS primarily induces G2/M cell cycle arrest in HK-2 cells treated with high glucose (Figure 8C).

## Discussion

Diabetic nephropathy (DN) remains a devastating microvascular complication of diabetes mellitus, representing a primary cause of end-stage renal disease (ESRD) globally. The pathogenesis of DN is multifaceted, involving intricate interplay between metabolic dysregulation, oxidative stress, chronic inflammation, and progressive fibrosis. Current therapeutic strategies, while offering some symptomatic relief, often fall short of halting or reversing DN progression and may be associated with long-term adverse effects. Consequently, there is an urgent and unmet need for safer and more efficacious therapeutic interventions that can target the underlying mechanisms of DN and confer sustained renal protection. Traditional Chinese Medicine (TCM), with its holistic approach and multi-component, multi-target nature, offers a promising avenue for managing complex chronic conditions like DN. Wulingsan (WLS), a classic TCM formula comprising *Polyporus umbellatus* (Zhu Ling), *Alisma plantago-aquatica* (Ze Xie), *Poria cocos* (Fu Ling), *Atractylodes macrocephala* (Bai Zhu), and *Cinnamomum cassia* (Gui Zhi), has a long history of use in regulating fluid metabolism and alleviating edema, a hallmark of DN. Modern pharmacological investigations have begun to unveil its diuretic, anti-inflammatory, anti-fibrotic, and anti-oxidative properties, lending support to its potential utility in DN management (Kang et al., 2021; Pang et al., 2021). However, a systematic understanding of the molecular mechanisms underpinning WLS’s therapeutic effects in DN has remained elusive, thereby limiting its broader clinical application. This study, therefore, employed an integrated approach combining network pharmacology, molecular docking, molecular dynamics simulations, and preliminary *in vitro* experiments to systematically elucidate the multi-component, multi-target, and multi-pathway molecular mechanisms of WLS in the treatment of DN.

This network pharmacology analysis identified active pharmaceutical ingredients from the five constituent herbs of WLS, which collectively targeted a multitude of proteins. Functional enrichment analysis of these targets revealed significant associations with key KEGG signaling pathways, most notably the PI3K-Akt signaling pathway and the MAPK signaling pathway, alongside critical GO biological processes pertinent to DN pathogenesis. Through rigorous PPI network construction and the application of multiple topological algorithms, we pinpointed five core protein targets: SRC, AKT1, TNF, ESR1, and HSP90AA1, which are likely central to WLS’s action in DN. Subsequent molecular docking studies demonstrated favorable binding affinities between key active WLS compounds and these core targets, and these interactions were further validated for their stability through 100 ns molecular dynamics simulations. Preliminary *in vitro* experiments using high glucose-treated HK-2 cells indicated that WLS could modulate cell viability and cell cycle progression, providing initial experimental support for its protective effects at a cellular level.

The PI3K/Akt signaling pathway plays a central role in regulating various cellular processes, including cell survival, proliferation, inflammation, fibrosis, and insulin resistance, all of which are disrupted in diabetic nephropathy. Activation of this pathway inhibits the activity of glycogen synthase kinase-3β (GSK-3β), increases the Bcl-2/Bax ratio, and reduces the release of cytochrome c from mitochondria into the cytoplasm. This sequentially suppresses the activation of caspase-3, ultimately leading to the inhibition of renal cell apoptosis (Wang et al., 2024; Wang et al., 2021). Aberrant activation or inhibition of this pathway contributes significantly to glomerular and tubular injury. Our findings suggest that WLS may exert its renoprotective effects, at least in part, by modulating the PI3K-Akt pathway, potentially through its interaction with core targets like AKT1. For instance, studies have shown that targeting the PI3K/Akt pathway can ameliorate podocyte injury and reduce ECM accumulation (Lu et al., 2019). Similarly, the MAPK signaling pathway, encompassing ERK, JNK, and p38 subfamilies, plays a pivotal role in mediating cellular responses to a variety of stressors, including hyperglycemia and inflammatory cytokines, which are characteristic of the DN microenvironment (Jubaidi et al., 2022). Dysregulation of MAPK signaling is implicated in podocyte apoptosis, mesangial cell proliferation, and the overproduction of extracellular matrix proteins, contributing to glomerulosclerosis and tubulointerstitial fibrosis (Chen et al., 2022). This network analysis indicates that WLS likely influences MAPK signaling, thereby mitigating these pathological processes. The ability of WLS to modulate these interconnected pathways underscores its potential as a multi-faceted therapeutic agent for DN.

Src family kinases are non-receptor tyrosine kinases involved in a myriad of cellular signaling events, including cell growth, differentiation, adhesion, and migration. In the context of kidney disease, SRC activation has been linked to pro-inflammatory and pro-fibrotic responses (Wang and Zhuang, 2017). Inhibition of SRC kinase has been shown to ameliorate diabetic renal fibrosis (Dorotea et al., 2022), suggesting that WLS components targeting SRC could interrupt these detrimental processes. As a key downstream effector of the PI3K pathway, AKT1 is central to cell survival, apoptosis regulation, and glucose metabolism. In DN, alterations in AKT1 signaling can contribute to podocyte dysfunction, glomerular hypertrophy, and tubular injury (Chen et al., 2022). Natural compounds, such as quercetin (present in some WLS herbs), have been shown to target AKT1 (Liu et al., 2025), providing a plausible mechanism for WLS’s action. Tumor Necrosis Factor-alpha (TNF-α) is a potent pro-inflammatory cytokine that plays a crucial role in the pathogenesis of DN by promoting renal inflammation, oxidative stress, and apoptosis (Guo et al., 2017). Elevated levels of TNF-α and its receptors are associated with the progression of DN (Niewczas et al., 2012). WLS, by potentially modulating TNF signaling, could alleviate the chronic inflammatory state in the diabetic kidney. Estrogen Receptor 1 (ESR1) has emerged as a significant player in metabolic health and kidney physiology. Estrogens, acting through ESR1, can exert protective effects against oxidative stress and fibrosis in the kidney (Ma et al., 2021). Dysregulation of ESR1 expression or function has been linked to an increased risk of type 2 diabetes and its complications (Gallagher et al., 2007). WLS’s interaction with ESR1 might contribute to its renoprotective effects, possibly by restoring a more favorable hormonal signaling balance within the kidney. Heat Shock Protein 90 alpha class A member 1 (HSP90AA1) is a molecular chaperone crucial for the stability and function of numerous client proteins, including many kinases and transcription factors involved in stress responses and signaling pathways. In diabetes, HSP90AA1 is implicated in managing cellular stress responses, particularly within the AGE-RAGE pathway, which drives chronic inflammation (Bellaye et al., 2014). Its expression has also been noted as a predictive marker in DN (Lu et al., 2015). By interacting with HSP90AA1, WLS may help to stabilize cellular homeostasis and mitigate stress-induced damage in the diabetic kidney.

Collectively, the modulation of these core targets and pathways by WLS translates into a multi-pronged therapeutic strategy against DN. The observed enrichment of GO terms related to inflammation, oxidative stress, apoptosis, and fibrosis aligns with the predicted interactions. WLS appears to orchestrate a beneficial shift by attenuating pro-inflammatory signaling through factors such as TNF and SRC, bolstering cellular survival mechanisms involving AKT1 and HSP90AA1, potentially counteracting fibrotic processes with targets like SRC and ESR1, and mitigating oxidative damage. Our preliminary *in vitro* data, showing WLS’s impact on HK-2 cell viability and cell cycle under high glucose conditions, provides initial experimental corroboration for these computationally predicted mechanisms, suggesting that WLS can directly influence renal cell responses to hyperglycemic stress. The broader immunomodulatory potential of TCM, as highlighted by studies on immune disorders in DKD, and the relevance of processes like ferroptosis and renal fibrosis further contextualize the multifaceted benefits WLS might offer.

### Limitations of the study

It is important to acknowledge that this study, while providing a comprehensive network pharmacology-based hypothesis and preliminary *in vitro* support, relies on predictions and initial experimental findings; therefore, *in vivo* investigations employing diabetic rodent models are imperative to validate the proposed mechanisms, determine pharmacokinetic parameters, and substantiate the overall therapeutic efficacy of Wulingsan in diabetic nephropathy. Furthermore, this study was confined to tubular epithelial HK-2 cells as a proof-of-concept model; inclusion of human mesangial cells and podocytes in future work will be necessary to more comprehensively delineate Wulingsan’s effects across all relevant renal compartments. Additionally, the current study did not perform quantitative chemical profiling to standardize the Wulingsan preparation used for *in vitro* experiments. While we used a commercially available formulation, future investigations will incorporate detailed compositional analysis and concentration reporting of key active constituents. This will enhance reproducibility, facilitate mechanistic interpretation, and support the translational relevance of Wulingsan-based therapies.

## Conclusion

In summary, this study systematically investigated the potential molecular mechanisms of Wulingsan in the treatment of diabetic nephropathy using an integrated strategy of network pharmacology, molecular docking, molecular dynamics simulations, and preliminary cell-based assays. Our findings reveal that WLS likely exerts its therapeutic effects through the synergistic action of its multiple active components on a network of core targets, including SRC, AKT1, TNF, ESR1, and HSP90AA1. These interactions, in turn, modulate key signaling pathways such as the PI3K-Akt and MAPK pathways, leading to comprehensive benefits against inflammation, oxidative stress, apoptosis, and fibrosis in the context of DN. This research provides a significant scientific rationale for the traditional use of WLS in conditions related to fluid imbalance and kidney dysfunction, and lays a foundational basis for its further clinical development and application as a novel therapeutic agent for diabetic nephropathy.

## Data Availability

The original contributions presented in the study are included in the article/supplementary material, further inquiries can be directed to the corresponding authors.
